# The Analysis of Selected Morphological and Hemodynamic Parameters of the Venous System and Their Presumable Impact on the Risk of Recurrence after Varicose Vein Treatment

**DOI:** 10.3390/jcm10030455

**Published:** 2021-01-25

**Authors:** Cezary Szary, Justyna Wilczko, Dominika Plucinska, Anna Pachuta, Marcin Napierala, Anna Bodziony, Michal Zawadzki, Jerzy Leszczynski, Zbigniew Galazka, Tomasz Grzela

**Affiliations:** 1 Clinic of Phlebology, 02-034 Warsaw, Poland; cezary.szary@klinikaflebologii.pl (C.S.); justyna.wilczko@klinikaflebologii.pl (J.W.); dominika.plucinska@klinikaflebologii.pl (D.P.); anna.pachuta@klinikaflebologii.pl (A.P.); marcin.napierala@klinikaflebologii.pl (M.N.); anna.bodziony@klinikaflebologii.pl (A.B.); michal.zawadzki@klinikaflebologii.pl (M.Z.); jerzy.leszczynski@klinikaflebologii.pl (J.L.); 2 Diagnostic Imaging Center MRI & CT, Center of Sport Medicine, 02-034 Warsaw, Poland; 3 Department of Radiology, Center of Postgraduate Medical Education, 01-813 Warsaw, Poland; 4 Department of General, Endocrine and Vascular Surgery, Medical University of Warsaw, 02-091 Warsaw, Poland; zbigniew.galazka@wum.edu.pl; 5 Department of Histology and Embryology, Medical University of Warsaw, 02-002 Warsaw, Poland

**Keywords:** venous insufficiency, morphological abnormalities, ovarian veins, parauterine veins, pelvic veins insufficiency, recurrence, renal veins, varicose veins treatment, venous system variants

## Abstract

Introduction: The current treatment of venous disease is focused on reflux elimination in main venous trunks, especially in the saphenous vein. However, a high recurrence rate, independent of the method of treatment, suggests that the reason of low effectiveness may be due to a strategy focused on symptoms, without considering their origin. Method: The aim of study was the comparison of retrospective data from 535 women with venous disease, either after treatment (*n* = 183) or not treated before (*n* = 352). The analysis concerned clinical symptoms and the results of the extended diagnostics, including the examination of the lower limb, pelvic and abdominal veins either using duplex-doppler ultrasound as well as venography with computed tomography or magnetic resonance. Results: The comparison of selected venous system parameters revealed more advanced disease progression in previously treated patients, compared to non-treated individuals (e.g., ipsi- or bilateral incompetence of sapheno-phemoral junction—29.5% vs. 20.4%, at *P* < 0.05 and 13.6% vs. 7.7% at *P* < 0.05, respectively). This difference could be explained by post-treatment alterations in the venous system, an older age and the higher number of pregnancies in the recurrence group. However, both groups did not differ in regards to the symptoms of pelvic venous insufficiency or the frequency of relevant variants/abnormalities in venous system. Conclusions: Based on the aforementioned findings, we postulate the revision of treatment strategy, which should consider abdominal and pelvic veins as the source of reflux in many female subjects.

## 1. Introduction

Chronic venous disease (CVD) is one of the most common clinical problems, which affects millions of people worldwide. Furthermore, the still-increasing prevalence of disease, especially pronounced in industrialized countries, is considered the consequence of lifestyle changes and an ageing population [1,2]. According to data from large-scale international survey, the Vein Consult Program, the presence of any symptoms and signs of CVD, including its early stages, was reported in 83% of adult population [3]. Although slightly varying in some details, including study design and the size of analyzed population, similar data were gathered from several cross-sectional population-based national surveys, including Edinburgh Vein Study, Bonn Vein Study, or the large multicenter study performed in a Polish population by the Jawien group [4,5,6,7]. Due to the chronic character of disease, its burdensome influence on patient’s life comfort, with swelling or venous eczema, high risk of thromboembolic complications or venous leg ulceration at most advanced stages, CVD at any stage should no longer be considered as a “cosmetic problem” only [2].

The treatment of CVD is focused on elimination of pathological reflux in affected veins using either surgical ligation and stripping or endovenous obliteration. The latter may be achieved by using various techniques, from minimally invasive non-thermal methods such as sclerotherapy, and cyanoacrylate-based chemical ablation, to semi-invasive radiofrequency- or infrared laser-based thermoablation [8,9,10,11]. The availability of less invasive and less painful methods of treatment within the last two decades increased the total number of performed procedures, but also noticeably changed their contribution to the market structure, with significant shift from surgery towards aforementioned less invasive endovenous techniques. Since new methods and devices are still introduced to the market, the knowledge or recommendation regarding which of them is better, safer and more effective, when compared to others, clearly has measurable value, especially when considering that the global varicose vein treatment market in 2018 was estimated for around 1.5 billion US dollars [12].

Regrettably, although all clinical trials concerning new methods initially provided enthusiastic results from short-term assessment [11,13], the vast majority of them have been disappointing when verified in long-term follow-up or when used in real practice [8,9,14,15,16,17]. The common clinical problem for all techniques is the unacceptably high rate of disease recurrence [14,18]. Apart from “the natural course of disease,” the treatment failure and recurrence of symptoms are usually considered to be operator- or method-dependent [8,9,15]. Unexpectedly, several randomized trials and meta-analyses have proved that the latter, although seems to have some impact on the local outcome, nevertheless has rather limited influence on the distant result of treatment, i.e., the risk of disease recurrence [14,17,18]. Obviously, the most important for the final result of such evaluation is the sufficiently rigorous definition of disease recurrence. When considering the recanalization of a treated vein or local neovascularization as the only manifestation of recurrence, without the broader context of venous system status, it allows to get a 100% success rate in the clinical study, but will certainly result in a rather poor outcome in long-term follow-up.

Interestingly, some recent observations may suggest that the main reason of such unsatisfactory long-term results may be a poor diagnosis, followed by qualification to suboptimal therapeutic intervention [19,20]. To verify this hypothesis, we compared data from the extended diagnostic protocol of the patients with CVD, either with recurrent disease, after its previous treatment or that were not treated before, in regards to selected morphological and hemodynamic parameters of their venous system.

## 2. Experimental Section

The retrospective assessment concerned data collected in the years 2017–2019 on the database of our clinic. The data originated from 2136 records, corresponding to consecutive patients, that were subjected to routine diagnostics and treatment procedures, according to our standard protocol. To improve the homogeneity of data, we aimed to exclude the role of sex-related differences [6]; therefore, the assessment was limited to the data of women only. The concept of the study was formally approved by the Local Ethics Committee at the Medical University of Warsaw (decision no. AKBE/181/2020).

In brief, our protocol comprised a two-steps diagnostic algorithm. Accordingly, in the first step, all patients were requested to answer several questions from a standardized CVD-oriented questionnaire, concerning the patient’s demography, current symptoms and general health status, concomitant diseases and previous treatments. Then, patients were subjected to a color duplex-doppler ultrasound scan of their venous system using a Toshiba Xario 100 diagnostic ultrasound system (TOSHIBA/Canon Medical Systems Co., Otawara, Tochigi, Japan) with an 8–14 MHz linear probe for the assessment of limb veins, and a 6–9 MHz convex probe for the examination of pelvic and abdominal veins. The examination of lower limb veins was always performed in standing position, and it was focused on the detection of the reflux (reversed flow >500 ms, spontaneous or induced by distal compression or Valsalva maneuver) and identification of its main entry and re-entry points in superficial venous system.

A clinically relevant reflux of a large vein, e.g., the great saphenous vein (GSV), anterior accessory saphenous vein (AASV), small saphenous vein (SSV) or their large branches, was recognized when it involved the segment located between at least two consecutive tributaries, independently of the entry point of reflux. The reflux in the sapheno-femoral junction (SFJ) was defined as a reflux from the common femoral vein (CFV) to the GSV through the incompetent terminal valve of the GSV, either with distal compression or with Valsalva maneuver. Any other reflux in the proximal GSV, with a competent terminal valve, hence, not propagating from the CFV, was considered as not related to SFJ incompetence [21,22]. The alternative entry sites for the reflux in the groin, propagating from either pubic, pudendal or epigastric veins to the superficial veins system of lower limbs, were assessed in inguinal and perineal points, in standing position, using the Valsalva maneuver. The origin of reflux in superficial veins on the posterior and lateral aspect of the thigh was assessed at a gluteal point [23].

The pelvic and abdominal veins were assessed while patients were in a supine and semi-sitting position. The transabdominal assessment concerned the morphology and blood flow in large veins, including the inferior vena cava (IVC), common, external and internal iliac veins (CILV, EILV and IILV, respectively) and both left and right renal veins (LRV and RRV). Particularly, the aforementioned vessels were screened to exclude their compression/obstruction. Furthermore, the diameter and blood flow in both the left and right ovarian veins (LOV and ROV, respectively), as well as in parauterine veins (PUV), were assessed.

The patients in whom any significant abnormalities were detected (or suspected), were subjected to the second step of protocol—a further detailed examination using the venography in computed tomography (CT-V) or in magnetic resonance (MR-V). The latter was the preferred method in women of childbearing potential and in patients with a known allergy to iodine or with unstable hyperthyroidism. The image acquisition protocols for both CT-V and MR-V were optimized to produce compatible data, as verified in three patients examined using both methods (data not shown). In both methods, the assessment was always performed with intravenous injection of the respective contrasting medium, using an automated syringe with a flow rate of 3.5–4 mL/s. The scanning area involved the venous system between the IVC confluence to the right atrium at the top, and the upper one-third of the thigh at the bottom. The analysis concerned the verification of previous findings from ultrasound scan and further extended the assessment of vein abnormalities. The standardized protocol concerned the measurement of selected veins diameter—always in the plane perpendicular to the vein axis, at the same location and in the same phase after contrast infusion. The assessment was particularly focused on the detection of morphological variability in venous system, as well as the presence of radiological features of pelvic venous insufficiency (PVI) [23,24,25,26,27,28].

The CT-Vscan was performed using 64-row, 128-multislice CT scanner Incisive (Philips, Best, the Netherlands). The image acquisition protocol concerned two venous phases — an early, approximately 50 s after achieving the saturation peak of Ultravist (or Iomeron) contrast agent in abdominal aorta, and the late venous phase, after 120 s.

The MR-V was performed using the MR scanner Ingenia 3.0T (Philips, Best, The Netherlands). In the first stage, the imaging was done without contrast enhancement in morphological sequences—T2, FatSat T2 and balanced turbo field echo (BTFE) gradient sequence. Then, after the injection of the ProHance gadolinium contrast, images were acquired in dynamic sequences consisting of up to six contrasting phases. Finally, the scanning of pelvic and vulvoperineal veins was performed with the delayed contrast enhancement using an mDIXON high resolution sequence.

The data from all aforementioned examinations were recorded on the database of our clinic and were the basis for decision regarding patients’ further treatment. In the present study, we have searched the clinical database using the following criteria: female, clinical symptoms of CVD—C1 to C4, according to widely used classification concerning clinical symptoms, etiology, anatomy and pathophysiology—CEAP [29], available complete data from abovementioned two-step protocol, i.e., including extended CVD diagnostics with CT-V or MR-V, no active thrombosis and no active malignancies. To maintain better homogeneity in the study group, patients with the most advanced CVD, classified as C5 and C6 according to CEAP, were excluded from the assessment.

From the initial 2136 records, all aforementioned criteria have been met in 535 records, which were then selected for the assessment. These records were further filtered using an additional differentiating factor—“previous treatment” and hence, they were subsequently divided into two groups—“Recurrence” (*n* = 183) and “No treatment” (*n* = 352). The randomly selected 25 records from both groups were subjected to the verifications of their accuracy by direct comparison with the source data. The verification concerned the direct comparison of alphanumeric data recorded in our electronic database (e.g., clinical scores, values from all vein measurements) with the original source of this data (clinical questionnaire forms, written reports from imaging, etc.). Based on full consistency with the source data, confirmed for each dataset selected for verification, we assumed a similar accuracy for the remaining records in the entire database.

The definition of “previous treatment” concerned any intervention in venous system, either surgery and/or any endovenous treatment, including sclerotherapy or thermoablation, performed before the patient attended our clinic and was subjected to the abovementioned diagnostic procedures using the two-step protocol.

Finally, both datasets were analyzed using either descriptive statistics or comparative assessment, with a Mann–Whitney U test, with *P* < 0.05 being considered statistically significant. In regards to the selected parameters, their association with a patient’s clinical status was evaluated using an odds ratio with 95% confidence interval (OR, 95% CI).

## 3. Results

The short characteristics of both analyzed groups in regards to their demography and main clinical features are shown in Table 1.

The initial assessment of the selected datasets revealed that patients that had undergone previous treatment were statistically significantly older and more frequently experienced leg pain or discomfort, as compared to the “No treatment” group. Additionally, the mean number of deliveries was higher in patients from the “Recurrence” group. Interestingly, the percentage of patients classified according to clinical symptoms, based on the CEAP classification, did not differ significantly among both groups.

According to the definition of “previous treatment,” all patients from “Recurrence” group were already subjected to the treatment with various methods in the past. These methods included surgical ligation and stripping, thermoablation (laser and/or radiofrequency) and sclerotherapy. The majority of patients (*n* = 159) were treated with a single method only, although some of them were treated several times.

Twenty-four individuals were treated with a combination of three or more various methods. Noteworthy, approximately 40% patients in the subgroups “surgery only” (*n* = 41) and “thermoablation only” (*n* = 15) were subjected to a single intervention. The remaining patients were subjected either to multiple treatments using the same procedure, or treated in combination with other methods.

**Figure 1:**
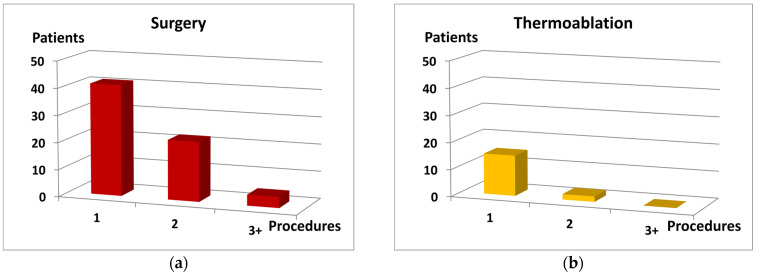

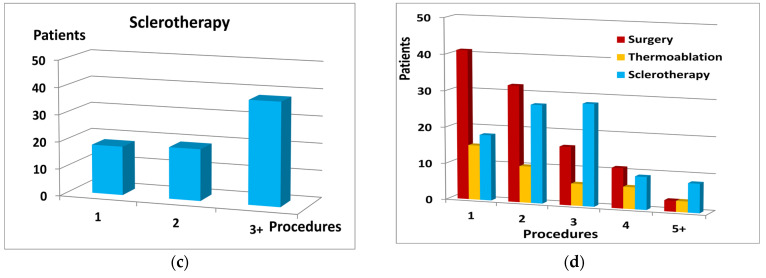
The number of procedures (horizontal axis) and distribution of the patient number (vertical axis) in regards to: (**a**) surgery as the only procedure; (**b**) thermoablation as the only procedure; (**c**) sclerotherapy as the only procedure; (**d**) each procedure alone or in combination with other methods.

The number of re-interventions varied among the analyzed methods, and was highest in “sclerotherapy only” group, where 36 patients were subjected to three or more sessions. Noticeably, in all methods, at least 80% patients experienced recurrence on the already treated limb, mostly in the same or nearby location. Further characteristics of the “Recurrence” group are shown in Figure 1 and Table 2.

Surprisingly, the comparative assessment of the results from the ultrasound examination showed that patients with recurrent disease, despite previous treatment, displayed statistically significantly more advanced morphological and hemodynamic features of CVD in their lower limb venous system. The main findings of the assessment were summarized in Table 3.

**Figure 2:**
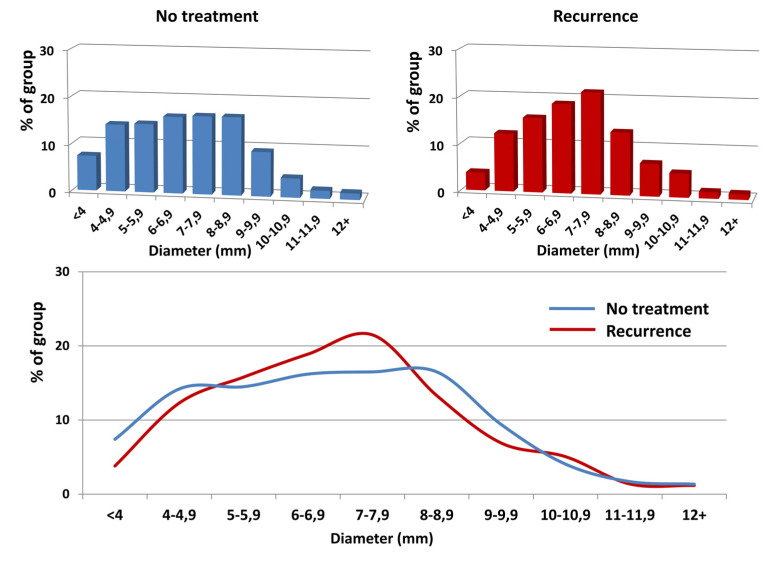
The distribution of left ovarian vein diameters (horizontal axis) in regards to their frequency (vertical axis) in the analyzed groups. The upper graphs show the distribution in each group; the lower graph shows the overlaid data from the upper graphs. Despite a marked shift in the prevalence of the LOV diameters towards patients with a diameter >6 mm in the “Recurrence” group, this difference appeared non-significant (OR 1.22; 95% CI 0.81–1.84; *P* = 0.33).

**Figure 3:**
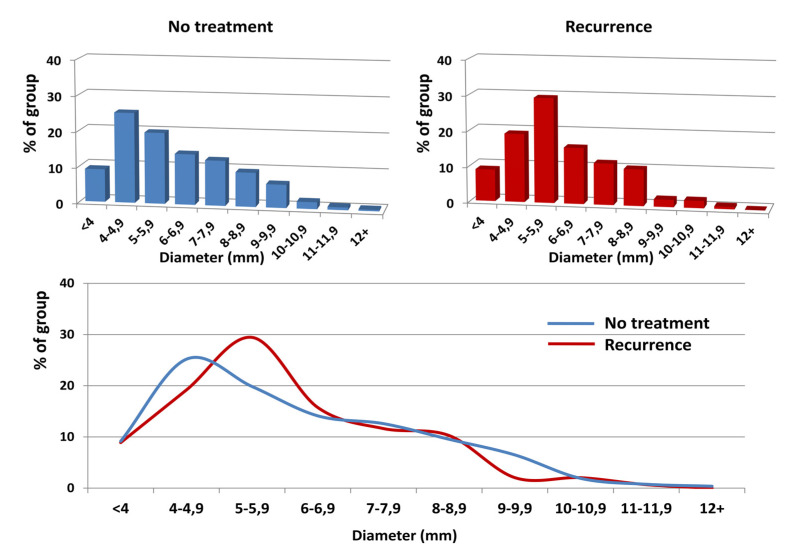
The distribution of the right ovarian vein diameters (horizontal axis) in regards to their frequency (vertical axis) in the analyzed groups. The upper graphs show the distribution in each group; the lower graph shows the overlaid data from the upper graphs. Despite a marked shift in the prevalence of the ROV diameters towards patients with a diameter >5 mm in the “Recurrence” group, this difference appeared non-significant (OR 1.34; 95% CI 0.86–2.08; *P* = 0.19).

The results of transabdominal ultrasound examination were verified in the second-step assessment either using CT-V or MR-V. Noteworthy, when compared to both advanced imaging methods, the accuracy of ultrasound scan was slightly worse, at least in regards to the underestimation of vein diameters or the detection of all coexisting morphological or hemodynamic abnormalities in each individual. Additionally, its sensitivity was lower in obese patients. Therefore, in some patients, the available ultrasound results were limited to detection of single abnormality only, which, however, was considered as sufficient indication to continue the extended diagnostic protocol. On the other hand, in all patients with recognized or suspected alterations in their abdominal and/or pelvic veins, the main findings from ultrasound examination were further confirmed in CT-V or MR-V. The most relevant observations from the second-step assessment were shown in Table 4 and in Figure 2 and Figure 3.

**Figure 4:**
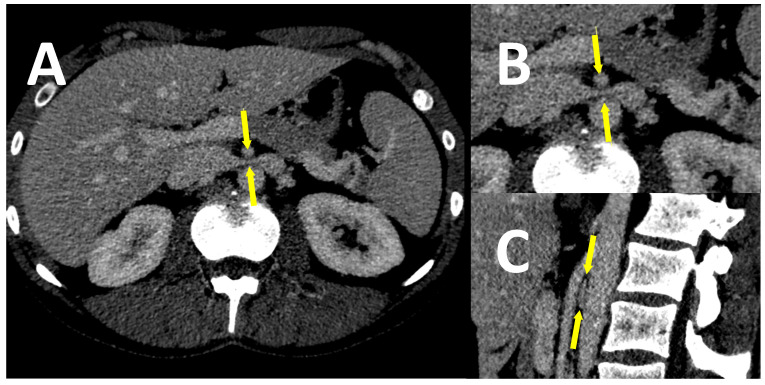
The images of a CT-V scan in a patient with LRV entrapment (“nutcracker” syndrome). LRV (marked with arrows) is compressed between the aorta and superior mesenteric artery (SMA), as shown on the transverse plane—whole image (**A**), enlarged image (**B**), and on the sagittal plane (**C**).

**Figure 5:**
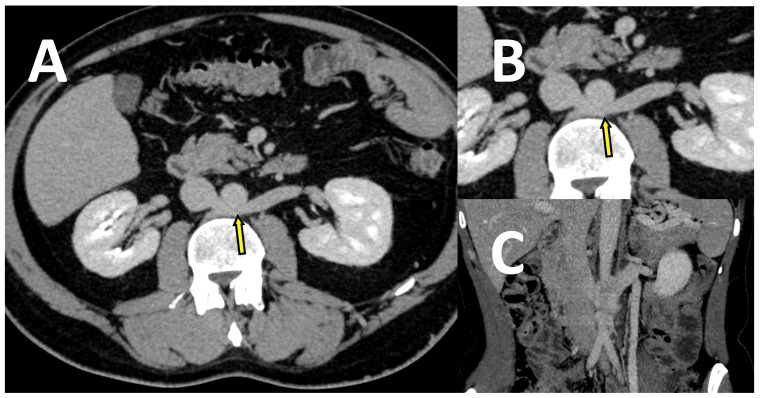
The images of a CT-V scan in a patient with a retroaortic location of LRV. LRV (marked with arrow) is compressed between the aorta and corpus of the lumbar vertebra, as shown on the transverse plane of whole (**A**) and enlarged image (**B**), or on the coronal plane (**C**).

**Figure 6:**
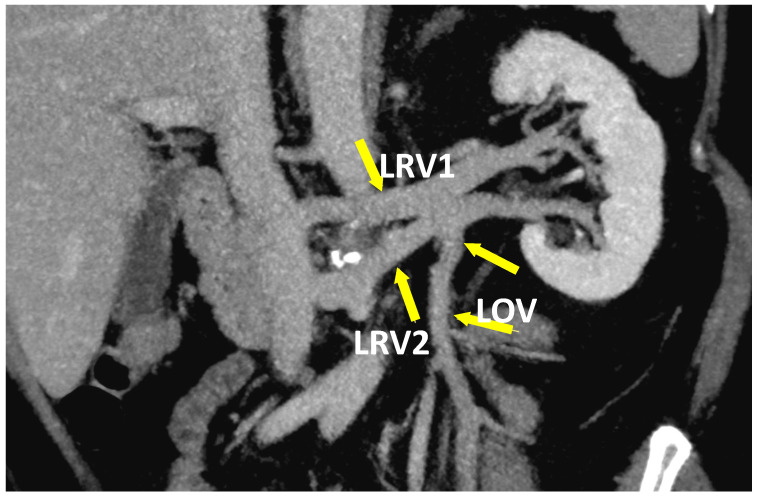
The coronal plane of CT-V scan in patient with duplicated LRV (LRV1 and LRV2). Blood from LOV is drained into LRV2.

Unexpectedly, the detailed analysis of CT-V and MR-V images has shown that almost one-third of individuals from both groups revealed clinically relevant (i.e., with evident morphological and hemodynamic consequences) anatomical variants with abnormalities of assessed abdominal and/or pelvic veins. Approximately one-fifth of them showed various variations in left renal vein anatomy. They included: significant LRV compression (entrapment) between the aorta and superior mesenteric artery (SMA), also known as the “nutcracker” phenomenon, retroaortic location of LRV, or retroaortic location of LRV branch draining the blood from left ovarian vein (Figure 4, Figure 5 and Figure 6, respectively).

**Figure 7:**
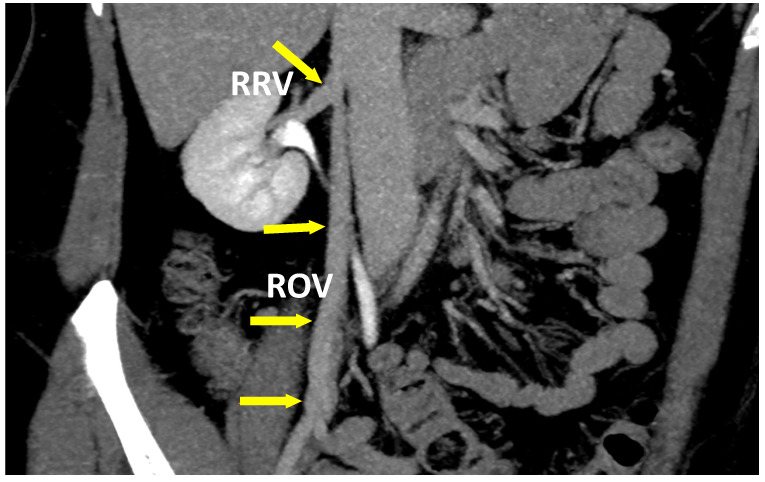
The coronal plane of a CT-V scan in a patient with atypical ROV outflow. Blood from ROV is drained into RRV.

**Figure 8:**
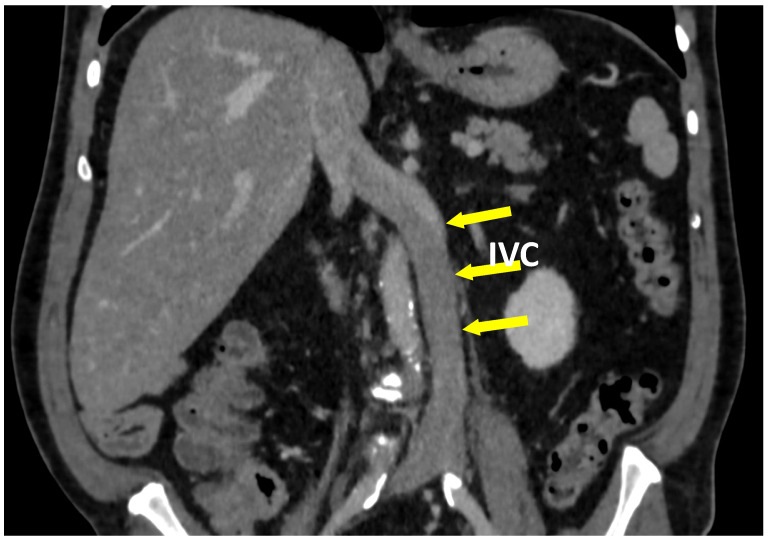
The coronal plane of a CT-V scan in a patient with left-sided inferior vena cava (IVC).

Subsequently, all abovementioned abnormalities were associated with various grades of overload and a reversed flow in LOV, followed by the development of collateral circulation. The other abnormalities, in decreasing frequency, concerned: various abnormalities of iliac veins (especially internal ILV), abnormal blood drainage from the right ovarian vein (Figure 7), and relatively rare abnormalities of inferior vena cava (Figure 8).

Interestingly, although the prevalence of LRV and LOV variations was visibly higher in the “Recurrence” group as compared to the non-treated patients, with a calculated odds ratio = 1.42 (95% CI = 0.80–2.51, at *P* = 0.23), this difference appeared to be non-significant.

**Figure 9:**
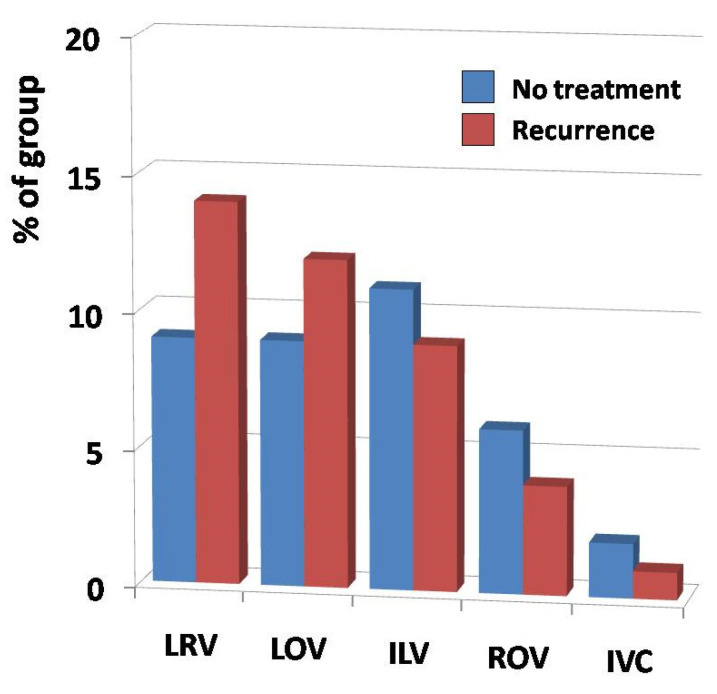
The prevalence of main anatomical variations and abnormalities (horizontal axis) in regards to their frequency (vertical axis) in analyzed groups. Abbreviations used: LRV—left renal vein, LOV—left ovarian vein, ILV—iliac veins, ROV—right ovarian vein, IVC—inferior vena cava.

Thus, none of the observed differences in the aforementioned variations reached statistical significance among both analyzed groups, although there was one exception. In six patients from the “Recurrence” group, a significant impairment of venous outflow, due to extensive neovascularization in the groin, was found. A short summary of these findings is shown in Figure 9.

## 4. Discussion

The reason of disease recurrence after varicose veins treatment is an unsolved riddle in surgery. The introduction of less invasive endovenous methods, including laser or radiofrequency thermal ablation as well as chemical ablation with cyanoacrylate adhesives or detergent-based foam sclerotherapy, was expected to improve the treatment effectiveness. In fact, when applied by experienced professionals, all these methods are characterized by lower risk of severe complications, as compared to surgical ligation and stripping [30,31]. Moreover, they may be performed without or with local anesthesia only. Hence, the minimally/less invasive methods allow early mobilization and fast patient return to daily and working activity; thus, their use is associated with a reduced risk of post-operative thrombosis [30,32,33]. Regrettably, despite the aforementioned benefits for the patient, when assessed in regards to the rate of disease recurrence, all these methods have substantially similar effectiveness in long-term assessment [14,16,17,18,34]. According to high quality data from large randomized trials and meta-analyses, the treatment efficacy of CVD in long-term follow-up is still highly discouraging. Since the evaluation criteria and the definition of recurrence differ among various studies, obviously, the rate of recurrence strongly depends on severity of criteria applied in assessment and, therefore, it ranges from 22 up to 55% in five-year observations [14,17,18]. Hence, it is noteworthy that, independently of the method, none of the aforementioned approaches was sufficiently effective to protect the patient against recurrence of CVD. Furthermore, as observed in 24 patients from our “Recurrence” group, even the combination of several various methods does not guarantee the long-term success and the avoidance of recurrence. Finally, when assessed in regards to some morphological and hemodynamic features, the current condition of leg vein system in patients from the “Recurrence” group was even worse, as compared to individuals not treated so far. Apart from statistically significantly higher prevalence of incompetent sapheno-femoral junction (either uni- or bilateral), involvement of two or more main venous trunks, or the presence of inguinal reflux entry point, the patients from the “Recurrence” group suffered from leg pain and/or discomfort more frequently as compared to non-treated individuals. One has to speculate that the more intense dysfunction of venous system with chronic leg discomfort/pain in “Recurrence” patients may result from post-treatment alterations (e.g., relevant neovascularization in the groin or significant anatomical and hemodynamic impairment in superficial venous system). The later constitute the real problem, especially in further approaches. Therefore, it is so crucial to improve the effectiveness of treatment, since each procedure introduces new alterations and, thus, it increases the level of difficulty and the challenge for the next operator.

On the other hand, all aforementioned symptoms or features could be considered as typical examples of disease progression, especially since patients from the “Recurrence” group were statistically significantly older compared to non-treated ones. However, although the frequency of patients with C3 and C4 stages, according to CEAP classification, was slightly higher in the “Recurrence” group compared to non-treated individuals, this difference appeared non-significant.

In that context, the explanation of treatment failure as “the natural course of disease”, although disappointing, seems to be the most logical answer. Thus, if the recurrence is actually the clinical manifestation of continuous disease progression, it is obvious that the previous treatment failed in regards to proper identification and elimination of the real origin of disease.

According to the current, or more precisely, predominant concept, based on the assumption that the disease is limited to the incompetent saphenous vein, the treatment of CVD is usually focused on the elimination of reflux in the venous trunk and, sometimes, in its main tributaries [8,9,14,18,35]. However, this strategy has clear limits when the origin of the reflux is located outside of GSV. Surprisingly, less than 30% patients in both groups from our study revealed valve incompetence in sapheno-femoral junction, whereas in more than 85% of patients, the upper entry points of reflux were identified in perineal connections of superficial venous system with pelvic veins. This observation is inconsistent with data from other studies, which reported up to 29.7% cases having a pelvic origin of reflux [36]. However, one has to take into account that our patients are not necessarily representative of the entire population, and patients with PVI could be over-represented in our study. On the other hand, this may suggest that, at least in those patients, the GSV incompetence may be the result rather than the cause of CVD [19,20]. It is plausible that the failure of the current CVD treatment strategy may be due to the misunderstanding of the hemodynamic pathomechanism of disease. This assumption could, at least partially, be supported by the long-term results of CVD treatment with ambulatory selective varices ablation under local anesthesia (ASVAL) procedure [37,38], and particularly a hemodynamic surgical approach CHIVA (from French: “cure conservatrice et hémodynamique de l’insuffisance veineuse en ambulatoire”, i.e., ambulatory conservative and hemodynamic treatment of venous insufficiency) [38,39]. When compared to surgical ligation and stripping, the CHIVA technique, based on ultrasound mapping and reflux re-distribution, was associated with the lower risk of nerve injury, and post-operative bruises, but with a higher risk of superficial vein thrombosis. Although the recurrence of varicose veins in long-term assessment was significantly lower after CHIVA, as compared to standard surgery, it was still very high (29.7% vs. 47.1%, respectively) [40]. Thus, even the hemodynamic approach of CHIVA did not identify or eliminate the reflux origin in almost one-third of the patients.

The explanation for that seems to be relatively straightforward. Although the worldwide golden standard for CVD investigation is duplex ultrasonography, usually, the examination is limited to the venous system of lower limbs only, without any attempt to assess the abdominal and pelvic veins [41,42]. The role of the latter, although still neglected by many authors, has been postulated as a key element in the CVD puzzle [19,20]. Noteworthy, more than 65% of our patients revealed the clinical and radiological features of PVI. Interestingly, the mean diameters of the LOV, ROV and parauterine veins were dilated, but they did not differ among either groups, despite the marked shift towards larger diameters (>6 mm for LOV and >5 mm for ROV) in the “Recurrence” patients, as observed in the detailed analysis of the diameter distribution. The possible explanation of that shift could again be older age, the significantly lower percentage of nulliparous women and the significantly higher mean number of deliveries in the “Recurrence” group, as compared to non-treated individuals [43]. Additionally, the occurrence of clinically relevant anatomical variants and/or abnormalities in abdominal and pelvic venous system did not differ significantly between both groups.

According to recent studies and the presented data, approximately 25–35% patients may reveal various anatomical abnormalities with a “collateral effect” in their abdominal or pelvic veins; however, the majority of cases result from pregnancy-induced overload and subsequent impairment of left ovarian and iliac veins axes [44,45,46,47]. Noteworthy, both anatomical variants and/or pregnancy-induced PVI may be the origin of perineal reflux, which, depending on the direction of its propagation, may result in the development of “atypical” varicose veins (usually located in lateral or posterior aspects of the thigh) or which, via the branches of the posterior accessory the saphenous vein (PASV), may contribute to the overload and subsequent insufficiency of the GSV.

In summary, some differences in the clinical condition of lower limbs and the occurrence of symptoms may result from post-operative alterations in venous system as well as the “natural course” of disease. On the other hand, all aforementioned morphological and hemodynamic similarities in abdominal and pelvic veins could be sufficient to conclude that both analyzed groups are almost identical in regards to the pelvic origin of CVD. Hence, it is plausible that, when undertaking the treatment in patients from the “no treatment” group, the use of the current approach, which neglects the pelvic source of reflux, will certainly result in outcome similar to that in the “Recurrence” group. Accordingly, to improve the long-term results of CVD treatment, we postulate to extend the basic examination protocol of the vein system by including the screening of the pelvic vein insufficiency. Furthermore, we indicate the necessity of the revision in the current strategy of CVD treatment, which should consider the pelvic and abdominal veins as the prevalent source of reflux in the leg venous system, at least in multiparous women [19,20,47]. When confirmed, PVI treatment should be considered as one of the due targets in that strategy. Nevertheless, although various approaches to manage PVI reflux have been proposed so far [19,20,21,48], since there is no consensus in regards to indications and contraindications for these procedures [49], they are still considered by many clinicians as controversial. Thus far, the most agreed indication to PVI treatment is the presence of pelvic symptoms, whereas the primary or recurrent varicose veins of lower limbs are usually not included as an indication for such treatment. However, since PVI represents the very frequent source of venous reflux in the lower limbs of multiparous women, apart from scleroembolization, the ultrasound-guided sclerotherapy of perineal refluxes should be considered as the key component of CVD therapy. Our preliminary results from the treatment designed and conducted in respect to the morphological and hemodynamic background of CVD, including the pelvic origin of venous reflux, are highly encouraging. Therefore, further studies focused on this issue are required.

## Figures and Tables

**Table 1:** Characteristics of study groups. The values shown in table represent mean (median) ± SD, or the number (percent) of patients reporting evaluated symptom in the group, respectively.

Parameter or Variable	Recurrence (*n* = 183)	No Treatment (*n* = 352)
Age	**45.5 (44.9) ± 9.9**	40.7 (38.9) ± 10.4 *
Number of pregnancies (P)	1.8 (2.0) ±1.3	1.7 (2.0) ±1.5
− P0	**34 (18.6%)**	98 (27.8%) *
− P1	41 (22.4%)	63 (17.9%)
− P2	65 (35.5%)	111 (31.5%)
− P3	32 (17.5%)	46 (13.1%)
− P4+	11 (6.0%)	34 (9.6%)
Number of deliveries	**1.7 (2.0) ± 1.2**	1.4 (1.0) ± 1.3 *
Predominant signs and symptoms:		
− dilated reticular and/or “spider” veins	75 (40.9%)	122 (34.6%)
− leg pain/discomfort	**104 (56.8%)**	148 (42.0%) *
− abdominal pain/discomfort	33 (18.0%)	71 (20.2%)
Clinical classification (CEAP):		
− C1	17 (9.3%)	39 (11.1%)
− C2	101 (55.2%)	213 (60.5%)
− C3	52 (28.4%)	76 (21.6%)
− C4	13 (7.1%)	24 (6.8%)

* Values marked with bold and asterisks are statistically significantly different among both groups (by Mann–Whitney U test, *P* < 0.05).

**Table 2:** Characteristics of the “Recurrence” group. The values shown in the table represent the mean (median) ±SD, or the number (percent) of patients in the group, respectively.

Parameter or Variable	Result
Mean (median) number of procedures per patient	2.1 (2.0) ± 1.2
Type of procedure with the frequency of the same location recurrence:	
− surgery (ligation and stripping):	103 (56.3%)
∗ the same limb/location recurrence within the subgroup	92 (89.3%)
− thermoablation (laser or radiofrequency)	40 (21.8%)
∗ the same limb/location recurrence within the subgroup	32 (80.0%)
− sclerotherapy	88 (48.1%)
∗ the same limb/location recurrence within the subgroup	82 (93.2%)

**Table 3:** The comparison of leg ultrasound examination results. The values shown in the table represent the mean ±SD, or the number (percentage) of patients with corresponding features, respectively.

Parameter or Variable	Recurrence (*n* = 183)	No Treatment (*n* = 352)
Number of main venous trunks with reflux	**2.02 ± 0.9**	1.75 ± 0.8 *
Incompetence in sapheno-femoral junction (SFJ)	**54 (29.5%)**	72 (20.4%) *
Bilateral incompetence in SFJ	**25 (13.6%)**	27 (7.7%) *
Detected entry points for reflux in the superficial system:		
− inguinal	**8 (4.4%)**	3 (0.8%) *
− perineal	157 (85.8%)	312 (88.6%)
− proximal (thigh) perforators	4 (2.2%)	9 (2.6%)
− distal (calf) perforators	3 (1.6%)	4 (1.1%)
Reflux in deep venous system	10 (5.5%)	22 (6.2%)

* Values marked with bold and asterisks are statistically significantly different among both groups (by Mann–Whitney U test, *P* < 0.05).

**Table 4:** The selected morphological and hemodynamic parameters of abdominal and pelvic veins found in second-step assessment with either CT-V or MR-V. The values shown in the table represent the mean (median) ±SD, or the number (percentage) of patients in the group, respectively.

Parameter or Variable	Recurrence (*n* = 183)	No Treatment (*n* = 352)
Diameter of LCILV (mm) ^(1)^	14.1 (14.5) ± 2.2	13.4 (13.5) ± 2.7
Diameter of LRV (mm) ^(2)^	8.1 (9.0) ± 3.8	7.8 (9.0) ± 3.6
Diameter of LOV (mm) ^(3)^	6.7 (6.5) ± 1.9	6.6 (6.5) ± 2.1
Diameter of ROV (mm) ^(3)^	5.8 (5.5) ± 1.7	5.9 (5.5) ± 1.9
Diameter of PUV (mm) ^(4)^	6.7 (7.0) ± 1.3	6.6 (6.1) ± 1.4
Reflux in LOV	125 (68.3%)	231 (65.6%)
Reflux in ROV	49 (26.8%)	112 (31.8%)

Abbreviations used: LCILV—left common iliac vein, LRV—left renal vein, LOV—left ovarian vein, ROV—right ovarian vein, PUV—parauterine veins; ^(1)^ measured below crossing with common iliac artery, ^(2)^ measured before crossing with superior mesenteric artery, ^(3)^ measured in lower (distal) 1/3 of the vein length, ^(4)^ the largest diameter observed.

## Data Availability

Not applicable.
